# The NHS Diabetes Prevention Programme: an observational study of service delivery and patient experience

**DOI:** 10.1186/s12913-020-05951-7

**Published:** 2020-11-27

**Authors:** Rhiannon E. Hawkes, Elaine Cameron, Sarah Cotterill, Peter Bower, David P. French

**Affiliations:** 1 Manchester Centre for Health Psychology, Division of Psychology and Mental Health, Univeraity of Manchester, Manchester, UK; 2 Division of Psychology, University of Stirling, Stirling, Scotland, UK; 3 Division of Population Health, Health Services Research & Primary Care, University of Manchester, Manchester, UK

**Keywords:** Type 2 diabetes, Diabetes Prevention Programme, Non-diabetic hyperglycaemia, Behaviour change, Intervention description, Intervention implementation, Patient experience

## Abstract

**Background:**

The NHS Diabetes Prevention Programme (NHS-DPP) is a nine-month, group-based behavioural intervention for adults in England at risk of developing Type 2 diabetes. Four independent providers were commissioned to deliver versions of the NHS-DPP, in line with NHS England specifications. This observational study maps NHS-DPP delivery in routine practice against the NHS specification, and compares service delivery with observed patient experiences.

**Methods:**

Researchers observed service delivery across eight complete NHS-DPP courses (118 sessions, median 14 sessions per course), consenting 455 participants (36 staff, 398 patients, 21 accompanying persons). Key features of NHS-DPP delivery were described using the Template for Intervention Description and Replication (TIDieR) framework. Researchers wrote detailed field notes during each session, including observations of patient experience. Field notes were content analysed; instances of positive and negative experiences were labelled and grouped into categories. Researchers used a novel method of comparing observed patient experiences to variations in programme delivery.

**Results:**

Delivery broadly followed NHS England’s specification and the plans set out by providers. Deviations included the scheduling and larger group sizes in some sessions. There was variation in the type and format of activities delivered by providers. Positive patient experiences included engagement, satisfaction with the programme, good within-group relationships and reported behavioural changes. Negative experiences included poor scheduling, large groups, and dissatisfaction with the venue. Where more interactive and visual activities were delivered in smaller groups of 10–15 people with good rapport, there were generally more instances of positive patient experiences, and where there were structural issues such as problems with the scheduling of sessions, poor venues and inadequate resources, there tended to be more negative patient experiences.

**Conclusions:**

Addressing issues that we have identified as being linked to negative experiences with the NHS-DPP could increase uptake, reduce patient drop-out and increase the overall effectiveness of the programme. In particular, modifying structural aspects of the NHS-DPP (e.g. reliable session scheduling, reducing group sizes, enough session resources) and increasing interaction appear particularly promising for improving these outcomes.

Type 2 diabetes is an international public health concern, in which global incidence increased to 422 million in 2014 [1]. Diabetes prevention trials in countries including China [2], Finland [3], United States [4], Japan [5] and India [6] have found lifestyle programmes to be effective in promoting behavioural change and reducing the incidence of Type 2 diabetes. Following international evidence, NHS England launched the NHS Diabetes Prevention Programme (NHS-DPP) in 2016; a behavioural intervention for adults in England who have elevated blood glucose levels, (i.e. non-diabetic hyperglycaemia), to slow or stop their progression to developing Type 2 diabetes [7]. The NHS-DPP is the largest diabetes prevention programme globally to achieve universal national coverage [8], thus, evaluations of NHS-DPP delivery are of particular value for the ongoing success of the programme and may inform other countries that are nationally rolling out health initiatives with multiple providers.

The NHS-DPP has been rolled-out in waves, gradually increasing coverage across England. Potential providers were required to propose a programme to meet NHS England specifications [9], based on evidence for diabetes prevention programmes to date [10]. NHS England stipulated the following features of service delivery: in groups of no more than 15–20 adults with non-diabetic hyperglycaemia, over at least 13 sessions, with the aims to achieve behaviour change to result in improved diet, increased physical activity and weight loss [9]. The programme was aimed at adults over the age of 18 years with an HbA1c of 6.0–6.4% (42–47 mmol/mol) or fasting plasma glucose level (FPG) of 5.5–6.9 mmol/l. Eligible patients were identified in primary care and referred to a local provider delivering the programme. Before enrolment onto the group sessions, patients were required to attend an initial assessment to introduce the programme, confirm their eligibility, and offered different times and locations for a programme in their local area, as well as collecting baseline measures.

During the third wave roll-out in 2018–2019, during which coverage of the NHS-DPP became nationwide, NHS England commissioned four independent provider organisations to deliver versions of the NHS-DPP, required to adhere to the programme specification [9]. A recent evaluation of the third wave of the NHS-DPP assessed the delivery plans (i.e. key intervention features) and behaviour change content planned by each provider, and reported that providers’ plans were generally in accordance with the NHS programme specification [11]. However, it is not currently known whether this planned programme delivery is being implemented in practice. Whether the four providers are delivering the NHS-DPP in line with their intervention plans is termed ‘intervention fidelity’, that is, whether an intervention is delivered as intended [12]. Accurate description of an intervention as actually delivered, rather than as planned, can increase transparency of intervention implementation, and potentially enhance the quality of interventions [13].

The Donabedian model [14] describes the quality of healthcare as being informed by its structure, process and outcomes. Structure describes the context in which healthcare was delivered (e.g. venues, equipment), process represents the transactions between patients and providers throughout healthcare delivery and outcomes represent the consequences of healthcare on the patients [14]. In line with the Donabedian model [14], this paper defines structural features of the NHS-DPP as venues, resources, session scheduling and equipment, process features are defined as the course content, activities and interactions within the group sessions, and the outcomes are considered as the observed patient experience of the NHS-DPP.

The best information currently available on NHS-DPP delivery is from an evaluation of the pilot NHS-DPP; a smaller-scale NHS-DPP intervention in 2015, before the programme began phased roll-out in 2016 [15, 16]. This evaluation [16] is currently the only study to report on patient experience of the programme which used qualitative telephone interviews, but the description is brief alongside data from other stakeholders such as commissioners. Nonetheless, key themes relating specifically to patient experience reported patients to benefit from the group support of sessions and positive behavioural changes made [16]. In the 2016 evaluation, providers spoke about tailoring the programme to local context [15, 16]. However, there is yet to be an evaluation of how this adaptation impacts on actual delivery of the NHS-DPP and whether the NHS-DPP can deliver comparable benefits to published trials in reducing the onset of Type 2 diabetes. Crucially, we do not know what structural and process features in the NHS-DPP are driving patient experience. Where multiple providers are delivering a complex multi-site programme, fidelity is likely to be lower [17], so it is important to understand variation in delivery between providers and sites.

Qualitative interview studies have the strength of eliciting in-depth views of participants, but tend to be sampled from few geographical sites and only include a willing sample of patients from each site. Gaining information on a broader sample of patients’ experiences of the NHS-DPP, based on observation rather than self-report, could provide valuable insight into the success of the programme. This could be of particular value for evaluating the first large-scale national multi-site diabetes prevention programme, as observations would capture the structure and processes of the intervention [14], which may be useful for the future success of the NHS-DPP and other large-scale public health initiatives going forward.

The main objectives were to: 1) describe the delivery of the NHS-DPP by the four providers, including discrepancies between what was planned and what was delivered and any variation in delivery between providers and sites and 2) describe patient experience of the NHS-DPP, as observed by researchers in the field. A secondary aim was to: 3) compare service delivery with observed patient experience.

## Methods

### Design and sampling

This study was part of a wider national evaluation of the NHS-DPP, described elsewhere [18]. We observed the delivery of the complete NHS-DPP course at eight sites across England between August 2018 and November 2019. Observing whole courses allowed researchers to understand the continuity of delivery across each programme. We observed complete courses at two sites per provider, with one site observed by EC and the other observed by REH. Sites were purposively sampled based on an overall sampling frame of NHS-DPP providers and sites in place during the evaluation period (2018–2019), with the aim of obtaining maximum variation in patient socioeconomic status (SES), ethnicity and geographical location with regards to urban and rural locations.

### Participants and consent procedures

The wider programme of research of which this study is a part of was approved by the North West Greater Manchester East NHS Research Ethics Committee (Reference: 17/NW/0426, 1st August 2017). Informed consent was obtained from all participants (patients, accompanying persons and facilitators) on the first day that researchers observed the NHS-DPP session, prior to the session starting and prior to researchers turning on the audio-recorder. It was explained that researchers were audio-recording and taking notes on the content of the sessions. Both the facilitators and patients were assured that their participation would remain confidential.

At four of the eight sites, group cohorts were merged during the second half of the NHS-DPP programme to accommodate for participant drop-out. Consequently, researchers consented a number of new participants at some of the sites. Before the beginning of each group observation, researchers checked that each participant had provided consent to taking part in the research. If there was a participant present who had not met the researcher, full written consent was obtained prior to audio-recording the session. If these new participants did not consent to taking part in the study, the group session was not audio-recorded and researchers attended a corresponding session at another location within the same site (e.g. if a new participant did not wish to consent during group session 11, researchers would attend another session 11 with a different cohort within the same geographical area for that provider to obtain data for that session). Different group cohorts were labelled ‘Group A,’ ‘Group B,’ etc.

### Materials

Observational data consisted of:

- Audio-recorded NHS-DPP sessions (*n* = 118), including seven initial assessments and 111 group sessions;
- Field notes using the Template for Intervention Description and Replication (TIDieR) structured framework [13], capturing service delivery information at each session. (See Additional file 1 for author-developed data collection form);
- Additional 1–2 pages of contemporaneous observational notes per NHS-DPP session. These captured views spontaneously expressed by participants, non-verbal aspects of delivery and any other notable observations.

Researchers attended all group sessions for each site, but were unable to attend an initial assessment consultation for one site with Provider A due to this provider ceasing to deliver face-to-face initial assessments during the data collection period. On four occasions where researchers EC or REH were unable to attend a session, another researcher from the wider team attended on their behalf. Consequently, less detailed observational notes were taken during these sessions, though researchers did discuss their observations with EC and REH following the session and notes were documented based on these discussions.

### Analyses

Service parameters from the TIDieR framework [13] (e.g. location, deliverer, group size, dose and scheduling, activities, materials, tailoring and fidelity) were extracted by REH from the audio-recordings and field notes and summarised for each site. This framework has previously been used to evaluate the NHS-DPP pilot sites providing telephone support in 2015, which described TIDieR as a useful tool for reporting interventions in applied healthcare research [19].

All field notes on observed patient experience for each session attended were compiled. Within each set of field notes experiences relating to course content, general engagement, venues, course access, other patients, facilitators and general feedback from patients were read thoroughly and extracted. Instances within the extracted pieces of text were given a label to succinctly capture the documented observation (example labels of instances: “good rapport built with facilitator”; “group support”). The labels of instances were grouped into categories to represent common positive and negative experiences observed by researchers (example category: “good group relationships between facilitators and peers”). The number of instances for each category were documented for each provider (e.g. “17 instances of category X observed with Provider Y across 26 sessions”). See Additional files 2 and 3 for the extracted data.

We made the decision to analyse the number of observed instances of patient experience throughout the courses, rather than analysing the number of sessions which included instances. Sometimes there was more than one instance of a category within a session which were unrelated (e.g. if patients had difficulty in finding the venue, and then later another patient stated that the venue was far away from their home, these were treated as two separate instances regarding the venue, but both occurred within the same session). As this was a content analysis, authors were not trying to quantify the number of sessions in which experiences occurred, rather, patterns in the data were analysed. Authors therefore analysed the number of instances for each category that were identified across provider courses, presented alongside number of observed sessions for each provider to give readers an indication of programme duration.

Each providers’ programme features identified using the TIDieR framework [13] were compared with the categories of patient experience to identify whether observed patient experiences corresponded to variations in providers’ programme delivery. We provide a qualitative assessment of associations on these links between delivery and experience, as the patient experience data were not suitable for inferential statistics and statistical analyses were not planned.

## Results

### Description of NHS-DPP delivery

A total of 36 facilitators, 398 patients and 21 family members consented to researchers attending, observing and audio-recording NHS-DPP sessions. Table 1 illustrates demographic characteristics at each site observed. The median Index of Multiple Deprivation (IMD) profile [20] for the eight sites was two, indicating generally high levels of deprivation, and ethnicity profiles according to site postcode [21] ranged from 15 to 96% white. Table 2 describes the service delivery features observed at each site.

**Table 1:** Demographic information of all participants consented during the NHS-DPP course observations at each site

	No. of facilitators	No. of patients	No. of family members	Median group size	SES profile (IMD)^a^	Ethnicity profile (% white)^b^
**Provider A**						
Site A_1_	5	86	3	12	2	15%
Site A_2_	2	95	2	14	2, 3^c^	75, 65%^c^
Provider A total:	7	181	5			
**Provider B**						
Site B_1_	6	43	2	17	2	45%
Site B_2_	6	23	3	15	3	96%
Provider B total:	12	66	5			
**Provider C**						
Site C_1_	2	52	3	12	6	91%
Site C_2_	7	34	2	10.5	1	54%
Provider C total:	9	86	5			
**Provider D**						
Site D_1_	5	37	4	8	2	65%
Site D_2_	3	28	2	6	2	88%
Provider D total:	8	65	6			
Overall consented:	36	398	21			

^a^*IMD, Index of Multiple Deprivation Scores associated with the lower super output area derived from venue postcodes, ranging from 1 (representing the 10% most deprived areas in England) to 10 (representing the 10% least deprived areas in England). Information obtained from Department for Communities and Local Government* [20]

^b^*Information on ethnicity for each geographical site was obtained from The Office of National Statistics* [21]*, taken from Census 2011*

^c^*Site A*_*2*_ *has two values for IMD and ethnicity profile as researchers attended two sites for the group observations*

**Table 2:** Description of NHS-DPP intervention delivery

Provider A		Provider B		Provider C		Provider D	
Site A_1_	Site A_2_	Site B_1_	Site B_2_	Site C_1_	Site C_2_	Site D_1_	Site D_2_
**What: Materials**							
Visual aids; posters; activity cards; worksheets; workbooks	Visual aids; posters; activity cards; worksheets; workbooks	PowerPoint; visual aids; exercise bands; workbooks	PowerPoint; visual aids; activity cards; exercise bands; workbooks	Pedometers; posters; visual aids; activity cards; workbooks	Pedometers; posters; visual aids; activity cards; workbooks	Visual aids; worksheets; external leaflets; workbooks	Visual aids; worksheets; external leaflets; exercise bands; pedometers; workbooks
**Materials in line with plans?**							
✓	✓	✘ not enough handbooks weeks 1–3; no PowerPoint in weeks 1–7	✓	✘ no pedometers until week 7; no weighing scales in sessions 6 and 12	✘ no workbooks in session 9; no weighing scales in session 10	✓	✓
**What: Procedures**							
Weigh-ins; goal setting; self-monitoring; barriers and solutions; government guidelines; sugar servings; food swaps	Weigh-ins; goal setting; self-monitoring; barriers and solutions; government guidelines; food swaps	Weigh-ins, goal setting; self-monitoring; fat models; quiz, barriers and solutions; food labelling; one-to-one reviews	Weigh-ins; goal setting; self-monitoring; fat, glucose and artery models; quiz; barriers and solutions; food labelling; one-to-one reviews	Weigh-ins; goal setting; self-monitoring; quizzes; barriers and solutions; government guidelines; carbohydrate and fat servings; food labelling	Weigh-ins; goal setting; self-monitoring; quizzes; barriers and solutions; government guidelines; carbohydrate servings; food labelling	Weigh-ins; goal setting; self-monitoring; barriers and solutions; government guidelines; sugar servings; food labelling; food swaps; one-to-one reviews	Weigh-ins; goal setting; self-monitoring; barriers and solutions; government guidelines; sugar servings; food labelling; food swaps; one-to-one reviews
**Who provided: Facilitator backgrounds**							
Public health; Nutrition; Psychology; Nutrition therapist; Teacher; Personal trainer	Personal training; Cardiac rehabilitation	Environmental science; Nutritional therapy; Sport’s science; Personal training	Nutrition & community health; Nutritionist; Nutrition; Sports nutrition; Sports & coaching	Sports health & nutrition; Nutrition	Health psychology; Teacher; Gym instructor; Mental health; Nutrition & health; Physical health & exercise	Personal training; Health sciences; Health trainer; Nutrition	Health promotion; Health psychology; Psychotherapist
**Who provided: Experience of facilitators delivering NHS-DPP (ranges)**							
0–29 months	2–12 months	4–12 months	4–24 months	2–12 months	0–19 months	1–36 months	3–13 months
**Group size (median)**							
12	14	17	15	12	10.5	8	6
**Group size in line with plans?**							
✓	✘ Some groups > 20	✘ Some groups > 20	✘ Some groups > 20	✘ Some groups > 20	✓	✓	✓
**Where**							
Community centre	Hotel; Leisure centre	GP surgery	Leisure centre	Community centre	Chapel hall; Charity building	Leisure centre	Community centre
**When and how much: Dose and scheduling**							
Sessions 3 and 4 delivered together; due to staff absence; maintenance sessions spaced 1–3 months apart instead of monthly	No. of sessions in accordance with plans; maintenance sessions spaced 1–3 months apart instead of monthly	No. of sessions in accordance with plans; 3-month gap before maintenance sessions	No. of sessions in accordance with plans; 2-month gap before maintenance sessions	No. of sessions in accordance with plans; Session 3 rescheduled after session 6 due to staff illness	No. of sessions in accordance with plans; Session 14 rescheduled the following month due to staff illness	No. of sessions in accordance with plans; 8-week instead of 4-week gap before maintenance sessions	No. of sessions in accordance with plans; Session 3 rescheduled due to staff absence
**Dose and scheduling in line with plans?**							
✘	✘	✓	✓	✘	✘	✘	✘
**Tailoring of intervention**							
Tailored to group demographic (e.g. discussions about Asian foods and lifestyles)	Tailored to group questions	Tailored to group questions; reviews tailored to individual; local services signposted	Tailored to group questions; exercise advice based on ability; reviews tailored to individual	Tailored to group demographic; exercise advice based on ability	Tailored to group questions	Tailored to group questions; reviews tailored to individual; local services signposted	Tailored to group questions; reviews tailored to individual; local services signposted
**Modifications to planned intervention**							
Session 7: BDA factsheet provided; Session 12: resistance exercises demonstrated only	Session 12: resistance exercises demonstrated only	No	Session 7: reduced gym memberships offered	Session 9: current news stories	Session 10: content missed due to staff absence	Session 13: British Heart Foundation ‘Eat Better’ booklet provided	Session 2: recipe books provided; Session 9: wellbeing leaflet provided

*The table headings correspond to the headings from the TIDieR framework* [13]*, with some adaptation from the researcher data collection form used in the field (see* Additional file 1*). In each table, providers are labelled A-D and the two sites observed for each provider are labelled 1 and 2 (*e.g. *Site A*_*1*_*, A*_*2*_*; B*_*1*_*, B*_*2*_*,* etc.*)*

*The number of group cohorts observed at each site are as follows: Site A*_*1*_ *= 3 cohorts; Site A*_*2*_ *= 3 cohorts; Site B*_*1*_ *= 2 cohorts; Site B*_*2*_ *= 1 cohort; Site C*_*1*_ *= 2 cohorts; Site C*_*2*_ *= 2 cohorts; Site D*_*1*_ *= 3 cohorts; Site D*_*2*_ *= 2 cohorts*

Results extracted using the TIDieR framework indicated that NHS-DPP delivery was generally in line with what was specified by NHS England in relation to session content, facilitators delivering the programme and the use of community venues for session delivery. Some discrepancies were observed between what providers planned to deliver and what they actually delivered (highlighted in Table 2). For example, in seven observed sessions (6%) providers A, B and C had more patients attending a group session (*n* > 20) than was stated in their delivery plans (maximum of between 15 and 20 patients). The scheduling of sessions were often discrepant, especially for Provider A, who had up to a three-month gap between their maintenance sessions, which were supposed to be monthly. Due to a supplier issue, Site C_1_ did not receive a delivery of pedometers until week seven instead of week one, which meant patients were unable to track their steps or report back any progress.

Activities included a mixture of education (e.g. the consequences of Type 2 diabetes, dietary and physical activity recommendations), group support (e.g. barriers and solutions to healthy living), knowledge testing (e.g. quizzes), visual activities (e.g. measuring the amounts of sugar and fats in food), and activities led by patients (e.g. collecting food packaging). The types of activities and their delivery varied across providers. For example, Provider A focused on group discussions and delivered visual activities via the use of posters and food models, Provider B delivered more educational activities, Provider C delivered quizzes to assess patients’ current knowledge about particular topics and Provider D had a focus on patients leading the session (e.g. collecting food packaging). All providers included group discussions in their delivery format, but Providers A and C accompanied these discussions with the use of worksheets and posters, whereas Provider B used PowerPoint and Provider D used external leaflets to accompany the session. Providers B and D signposted to local services at both sites observed.

There was some variation across sites regarding the tailoring and modifications of the programme. Sites A_1_ and C_1_ in particular tailored session content to the group demographic. For example, site A_1_ included discussions about Asian foods and lifestyles and site C_1_ tailored information for an older age group. Other sites only tailored the intervention in response to group conversations. Sites A_1_, D_1_ and D_2_ handed out a number of additional leaflets to supplement the session content. On occasions where a session had to be cancelled, sites C_1_, C_2_ and D_2_ rescheduled their sessions, whereas site A_1_ covered the content of two sessions within one session. During one session in site D_2_ when there was a low turn-out of patients, content was delivered at the following session when more patients were present.

### Description of observed patient experience

Field note content analysis yielded 127 instances of positive experiences and 83 instances of negative experiences observed across the 118 sessions attended. From these observed instances, three categories of positive experience and three categories of negative experience were identified. Table 3 highlights some of the key features of each of the provider programmes (identified using the TIDieR framework [13]), alongside the number of instances of positive and negative patient experiences observed per category across providers. Table 4 provides examples of both ‘positive’ and ‘negative’ instances of patient experiences that were documented in researchers’ field notes. (See Additional files 2 and 3 for all documented positive and negative patient experiences, extracted from observational notes).

**Table 3:** Provider programme characteristics and number of instances of positive and negative patient experience observations

	Provider A	Provider B	Provider C	Provider D
**Features of provider programme**				
Activities	Interactive, visual	Education-based	Interactive	Patient-led
Materials	Worksheets, posters, activity cards, food models	Workbooks, use of PowerPoint	Workbooks, posters, activity cards	Workbooks, additional leaflets provided
Group size	Generally groups of 10–15 people	Generally groups > 15 people	Generally groups of 10–15 people	Generally groups < 10 people
**Instances of positive patient experiences observed**^a^				
High engagement and satisfaction with the programme	17	13	19	10
Good group relationships (facilitators and peers)	21	8	15	7
Patient behaviour changes	5	2	2	8
Overall no. of positive experiences	43	23	36	25
**Instances of negative patient experiences observed**^a^				
Scheduling and size of group sessions	14	3	16	8
Factors influencing disengagement / dissatisfaction in session	2	10	11	4
Venue	2	2	8	3
Overall no. of negative experiences	18	15	35	15

^a^*The number of sessions observed for each provider are as follows: Provider A = 26 sessions; Provider B = 26 sessions; Provider C = 38 sessions; Provider D = 28 sessions*

**Table 4:** Positive and negative patient experience categories observed in NHS-DPP delivery

	No. of instances^a^	Examples from observational notes
**Positive patient experience**		
High engagement and satisfaction with the programme		
All sites	59	“The mindfulness activity was very popular with the group and some service users asked to do this activity again at the end of the session …”
		“The service user gave very good feedback on the programme, she said she hoped it would continue and that everyone would get as much out of it as she had; she said the main thing she had learned was knowledge about what to eat and what to avoid.”
Good group relationships between facilitators and peers		
All sites	51	“[Facilitator] was very engaging in the way he delivered the session. All the service users got involved with the discussion and asked questions. [Facilitator] seemed to build a rapport with the group very quickly.”
		“The group works well together, good relationships between service users, good peer support (e.g. congratulating each other if lost weight at start of the session).”
Patient behaviour change		
All sites	17	“One woman had managed to do 8000 steps every day this week, had even done 13,000 one day, and had walked 45 min home from the shop one day – sees the group as worthwhile.”
		“One man said he had lost 9 kg and his family commented on how much weight he had lost, but he felt very healthy and strong; one man said he would carry on with what he had learned, as he had been encouraged to do more exercise; he had made most changes in the first period of the course, but had managed to maintain it.”
**Negative patient experience**		
Scheduling and size of group sessions		
All sites	41	“Two service users complained about the lack of notice for this session – one lady was only given notice at 5:30 pm yesterday afternoon and another man was given notice at 9 pm yesterday evening and he had to cancel some plans in order to attend the session today.”
		“Difficult to manage the group with so many people attending; had to split the group into two for two activities, however even half the group couldn’t all fit around the activity table; lots of talking so difficult to hear all of the conversation and not everyone gets a chance to join in.”
Factors influencing disengagement / dissatisfaction within the session		
Sites A_1,_ B_1,_ B_2,_ C_1,_ C_2,_ D_1,_ D_2_	27	“This session was very heavy going – for over an hour there was information about very serious health consequences and risks of type 2 diabetes, with no activities to break it up; by the time they had a break people were commenting on “brains bursting.””
		“Some service users had difficulty opening up the pedometers to read the screen. Some pedometers seemed to be faulty as they would not re-set so [Facilitator] took those ones back in.”
Venue		
Sites A_1,_ A_2,_ B_1,_ C_1,_ C_2,_ D_1_	15	“Attendees said [venue] was hard to find (not well-known or well sign posted).”
		“For one woman, attending the class is a “five hour round trip” as it takes two buses/ one hour to get there and get home.”

*Extracted texts are presented as they were typed by the researcher after each session observation.*

^a^*Out of 118 observed sessions*

Generally, patients demonstrated engagement and satisfaction with the programme (*n* = 59 instances extracted from observational notes of 118 sessions). Researchers observed engagement with activities and discussions, and patients expressed enjoyment of mindfulness and visual activities in particular. Patients also reported general satisfaction with the NHS-DPP (e.g. telling others about the programme, learning about healthier foods). Good relationships within the groups were observed (*n* = 51 instances extracted from observational notes of 118 sessions). For example, facilitators built a good rapport with their groups and peer support was noted, such as meeting each other outside of the programme, giving suggestions or advice to each other and sharing honest accounts and experiences. Patients also reported positive behavioural changes made (*n* = 17 instances extracted from observational notes of 118 sessions), including increasing daily steps, learning new recipes and corresponding weight loss.

However, a number of notable negative experiences were observed and documented by researchers. There were observed structural issues with the scheduling and size of group sessions (*n* = 41 instances extracted from observational notes of 118 sessions), some of which included incorrect session dates and times provided, oversubscribed sessions, problems with text reminders, future session dates not confirmed and cancelled sessions not communicated. There were observed factors influencing disengagement or dissatisfaction within the session (*n* = 27 instances extracted from observational notes of 118 sessions), including patients disengaging with activities (e.g. too much complex information, difficult activities, room layout), issues with the session resources (e.g. unable to provide resources, issues with pedometers) and general patient dissatisfaction or feedback from the session. For example, one patient reported that they would have liked more demonstrations and practical sessions on cooking healthy meals. There were structural issues reported with some of the site venues (*n* = 15 instances extracted from observational notes of 118 sessions), such as the rooms being too hot in temperature, patients having difficulty finding the room or venue, access issues, distance of venue from patients’ homes and noise disruption.

### Relationships between patient experience and provider delivery

Providers A and C had more instances of positive patient experience regarding engagement and satisfaction with the programme and more observed positive relationships within their sessions documented in researcher field notes. (See Table 3 for numbers of instances observed and the number of sessions observed across each provider). Assessment of the key features in their programmes show that these providers had group sizes of generally between 10 and 15 people and delivered more interactive and visual activities with the use of worksheets, posters and food models. Provider D which delivered more patient-led activities (e.g. collecting food packaging) had more instances of observed behavioural changes in comparison to the other providers.

Regarding observed negative patient experiences, Providers A and C had more instances of reported issues with the scheduling of sessions compared to Providers B and D. These included being unable to confirm future session dates and incorrect session dates and times provided. A higher number of instances influencing dissatisfaction and disengagement within sessions was observed for Providers B and C. For example, such instances were documented when patients reported complex information difficult to understand, issues with session resources (e.g. not enough handbooks) and group sizes > 15 people. Provider C had more reported issues with the venue compared to other providers.

## Discussion

Our observations suggest that NHS-DPP delivery was generally in line with the NHS service specification [9] with regards to the session content and processes of the NHS-DPP, according to the data extracted using the TIDieR framework [13]. Researcher field notes indicated positive patient experiences, as well as negative patient experiences in relation to the structure of the NHS-DPP. There are significant organisational differences and modes of delivery which appear to have generated both positive and negative responses from patients of the NHS-DPP. In particular, there appeared to be more positive patient experiences observed within sessions (e.g. group rapport, engagement with interactive activities) and more negative patient experiences appeared to be linked to structural issues (e.g. session scheduling, group sizes, the venue, and issues with resources). More instances of positive patient experiences were observed when provider programmes had more visual and interactive activities, delivered in groups of 10–15 people. Thus, there are some improvements which, if addressed by providers, may improve the overall running of the programme.

### Strengths and limitations

To our knowledge, this is the first paper describing NHS-DPP delivery, including structural features, within-session process features and observed patient experience. We were able to observe the whole NHS-DPP course at each of the eight sites and observed a wide range of facilitators with varying experience and backgrounds. All sessions were audio-recorded, a ‘gold standard’ for fidelity evaluations [22], enabling a more detailed data extraction of the programme delivery. The use of the TIDieR framework [13] allowed for the complexities of delivering such an intervention in practice to be transparently documented.

This paper has provided a novel way of assessing patient experience, utilising in-depth observational notes across a much larger sample than would have been possible with qualitative research. Thorough observational notes were written (up to two sides of A4) per session. However, the patient experience described in this paper was that observed by researchers and documented in their notes, thus, observed patient experience was researchers’ interpretation. Only the corresponding field notes for each session were analysed to assess observed patient experience, as the audio recordings further presented over 200 hours of data. However, researchers’ observational notes were able to capture occurrences within sessions and non-verbal aspects of delivery which would not have otherwise been captured on the audio recording (e.g. informal conversations with patients, group interactions during activities). The use of researcher field notes are not often used to analyse or present data, however, we have found this a useful method to provide an in-depth analysis of what happened within sessions, especially with regards to the structural issues observed. These are valuable insights into the running of a national programme and may contribute to its future success in ensuring that the intervention continues.

Researchers were only able to observe and document volunteered views in their field notes, thus the data presented is naturally occurring data. For example, if a particular observation was not documented in researchers’ field notes for that session (such as high engagement), it does not mean it did not happen in that session. Further, researchers could only observe and interact with patients who continued attendance at the NHS-DPP. Consequently, this study cannot provide insights into barriers of attendance; a limitation also present in previous research on patient experience of the NHS-DPP [16]. Despite not having any formal estimation of observation reliability, researchers discussed what should be included in the observational notes beforehand, and both researchers documented similar types of observations in their notes (see Additional files 2 and 3). Further, despite having single observers at the sessions, both researchers each attended the delivery of a whole programme for each of the four providers delivering the NHS-DPP.

Although we attended the full NHS-DPP course at eight sites across England, the sample of eight geographical sites is still small, yet all that was feasible, given the length of the NHS-DPP programme (9–12 months), and the resources required for intensive observation. Further, we cannot be sure from these observations whether the issues identified are related to the particular courses we observed, or systemic issues that reflect the way NHS-DPP providers organise and run their courses. However, we have been able to identify associations between providers’ programme characteristics and observed patient experience at the eight sites attended.

Despite this, our purposive sample sought to assess diversity in NHS-DPP settings; researchers aimed to sample sites with as much variation in SES, ethnicity and geographical location as possible and 455 participants were consented. This approach has additional advantages to qualitative research examining patient experience, which gathers in-depth views from a smaller number of participants, usually somewhat restricted by geographical site. Our study includes observational data rather than self-report, thus does not have the same reporting biases. Although, it could be argued that the observed patient experience is not as direct as gathering views on patient experience in interviews. Nonetheless, this study provides a description into the patient experiences observed in the NHS-DPP, in which the analysis has the advantage of identifying broad patterns in the data.

### Relation to existing research

The only previous research to explore patients’ experiences of the NHS-DPP was during the pilot phase [16]. However, this interview study was conducted before the NHS-DPP was implemented nationally, patient data were analysed alongside data from other NHS-DPP stakeholders, and the comparison of patient experiences across sites and providers was not within the previous evaluation’s scope. Nonetheless, our observations are in accordance with those findings, as positive relationships were observed within groups, both with other peers and facilitators, and patients reported positive behavioural changes made to their lifestyles. The current study has further highlighted two new findings relating to the delivery of the NHS-DPP; the use of interactive and visual activities within NHS-DPP sessions (process features) appear to enhance patient experience, but issues with structural features of the programme such as session scheduling, group size and issues with session resources appear to impact negatively on patient experience.

Whilst previous qualitative studies on patient experiences of other behavioural interventions have provided an in-depth insight into interpersonal factors and participant motivations [23–25], they have not fully addressed the structural factors which are also important for the successful implementation of an intervention. Although the qualitative literature did allude to the fact that having the programme sites in more accessible locations with greater flexibility in session times and days could further improve patient experience [23–25], this has not been extensively researched. However, such structural features can have a direct influence on the processes and outcomes in programmes, for example, if there is insufficient resources and equipment or sessions cannot be scheduled, this can prohibit patients from accessing the support they require from the programme [26]. As found in the present study, the scheduling of sessions, group sizes, issues with provider resources and site venues were all structural issues which appeared to negatively affect patient experience. Recent data published on the early outcomes of the NHS-DPP highlight that course completion differed between providers [8]; upon comparing our data with this early outcome data, it appears that the providers with the lowest course completion rates [8] had more scheduling issues observed in the current study.

### Implications for practice

We observed disengagement within the sessions when patients reported information was difficult to understand, when there were issues with obtaining session resources, and when group sizes were greater than 15 people. Although we cannot be certain whether these are systemic issues in the way NHS-DPP providers are delivering their courses, our findings suggest that delivering more interactive activities with less complex information and having enough resources to supplement the session content may enhance patient experience. Given the NHS-DPP is the first national roll-out of a diabetes prevention programme to ever be implemented in routine practice, our findings may be of great value to make improvements to future waves of the programme, or for commissioners of other public health initiatives.

Despite the NHS-DPP being a national programme, there is variation in how the intervention is delivered by providers. There are clearly some deviations from providers’ protocols and what was specified by NHS England [9], but we do not know whether this variation is also present in other behavioural programmes. The importance of tailoring the intervention content according to the group demographics if often argued, known as adaptation in form (e.g. variation within providers according to local context) [17, 27]. However, other types of variation between providers (e.g. group sizes) may be explained by pressures faced by providers such as waiting lists to get onto the course, people wanting to switch courses, local insights of ‘did-not-attend’ rates, or incentives for commercial providers to enrol patients onto the NHS-DPP. Such variation is not adaptation, but suggests drift from the original NHS Service Specification [9], especially at the structural level with regards to the scheduling of sessions and group sizes. Too much drift from the specification will result in the NHS-DPP not being delivered with fidelity to the evidence base, and it is unclear how that would impact on effectiveness. Such structural issues observed highlight the wider issues of rolling out a national programme. The NHS-DPP started with seven small pilot sites in 2015 and rolled out to a multi-site programme in 2016, with commercial providers under pressure to deliver results with limited capacity across large geographical areas.

### Implications for research

Now the NHS-DPP is in the fourth year of implementation, some of these structural issues may have since been improved, although this is not certain. There is now a fifth provider commissioned to deliver the NHS-DPP alongside the other four providers; further observations of the NHS-DPP in the field would be beneficial to establish whether these structural features (e.g. session scheduling) remain an issue, or whether such issues are only present in the early stages of programme implementation. Further, it would be useful to replicate and advance this method using two researchers at each site observation and formal reliability testing, which would provide wider applicability of the current model used.

Future research could also examine the impact that different facilitator characteristics may have on the outcomes of the NHS-DPP. We observed 36 facilitators from diverse backgrounds with varying levels of experience and facilitating styles. Our observations suggested that good relationships with facilitators were linked with positive patient experience, but it is not clear which features of facilitators or training best brings this about. Further, we do not know the impact of the therapeutic relationship between the facilitator and the group on learning and retention of the NHS-DPP. Qualitative interviews with facilitators about their views and experiences of delivering different aspects of the NHS-DPP would give insight into additional requirements for facilitators going forward. Lastly, the authors of the present study have also assessed fidelity of delivery of behaviour change techniques in the NHS-DPP which is described in a separate publication [28].

## Conclusions

Overall, we observed positive instances of patient experience such as engaging with the overall programme, providing peer support, developing good relationships with facilitators and making positive behavioural changes. Such experiences were observed more often in programmes containing interactive and visual activities, delivered in groups of 10–15 people in line with the programme specification. Our observations of negative patient experience, in particular concerning the scheduling and size of group sessions, are improvements at the structural level that may further improve the delivery of the NHS-DPP and its longer-term success. By addressing these issues we have identified as being linked to negative patient experience, this could increase the uptake, reduce drop-out and increase overall effectiveness of the NHS-DPP.

## Supplementary Information

**Additional file 1:.** Observation of DPP Delivery Groups & Initial Assessments TIDieR Data Collection Form.

**Additional file 2:.** Positive patient experiences, extracted from observational notes.

**Additional file 3:.** Negative patient experiences, extracted from observational notes.

## Data Availability

All observational notes on patient experience analysed during this study are included in this published article and/or supplementary information files. The service delivery dataset generated and/or analysed during the current study are not publicly available due to confidentiality agreements with the provider organisations, as some information is commercially sensitive. Some datasets are available from the corresponding author on reasonable request, although authors will require the explicit permission of the relevant provider organisations.

## Abbreviations

NHS-DPP: National Health Service Diabetes Prevention Programme
TIDieR: Template for Intervention Description and Replication framework
