# Supplementary material for: The NHS Diabetes Prevention Programme: an observational study of service delivery and patient experience
Source: BMC Health Serv Res. 2020 Nov 27;20:1098. doi: 10.1186/s12913-020-05951-7 (PMC7694420; doi:10.1186/s12913-020-05951-7)
Supplement: Supplementary file 1 — Additional file 1:. Observation of DPP Delivery Groups & Initial Assessments TIDieR Data Collection Form. [file 12913_2020_5951_MOESM1_ESM.docx]

**Additional File 1. Observation of DPP Delivery Groups & Initial Assessments
TIDieR Data Collection Form**

File number:

**Researcher: ………………………………………………………………………………………………………**

**Date of observation: ………………………………………………………………………………………………………**

**Provider organisation: ………………………………………………………………………………………………………**

**Location of delivery session: ………………………………………………………………………………………………………**

**Recorder: ………………………………………………………………………………………………………**

**File number on recorder: ………………………………………………………………………………………………………**

**Time (start/end): ………………………………………………………………………………………………………**

**Duration: ………………………………………………………………………………………………………**

| **Data** | **Comments** |
| --- | --- |
| **Venue**  Type of venue:  Venue/ room details: |  |
| **Facilitator delivering session**  Length of time delivering DPP:  Length of time since training:  Professional background:  Gender: |  |
| **No. and gender of service users**  Service users (M/F):  Support persons (e.g. family, carers): |  |
| **Aims & objectives of course or session explained?** (Y/N): |  |
| **Materials**  Describe physical or informational materials given out or used in delivery |  |
| **Tailoring**  Describe personalisation or adaptation (what, why, when, how) |  |
| **Modifications**  Describe changes to planned intervention (what, why, when, how) |  |
| **Fidelity**  Describe if provider evaluates fidelity (e.g. quality assessment) |  |
| **Any other information/comments** |  |
