# Supplementary material for: The NHS Diabetes Prevention Programme: an observational study of service delivery and patient experience
Source: BMC Health Serv Res. 2020 Nov 27;20:1098. doi: 10.1186/s12913-020-05951-7 (PMC7694420; doi:10.1186/s12913-020-05951-7)
Supplement: Supplementary file 2 — Additional file 2:. Positive patient experiences, extracted from observational notes. [file 12913_2020_5951_MOESM2_ESM.docx]

**Additional File 2. Positive patient experiences extracted from observational notes, corresponding to each category**

| **Sub-category** | **Sites** | **No. of instances observed** | **Extracted from observational notes** |
| --- | --- | --- | --- |
| **Category 1: High engagement and satisfaction with the programme [n = 59 instances]** | | | |
| High engagement (n = 43) | Sites 1, 2, 3, 5, 6, 7 | Site 1 = 15 | Engagement during the session seemed high – as this was an elderly group they seemed to have already been affected by diabetes in some way (e.g. other friends/family members) so many of them seemed motivated and grateful for the session.  Engagement was high again and many were asking questions about different types of snacks and what is better to consume.  High engagement – many service users actively asked questions and they all participated in activities and quizzes.  All members engaged in the activities and asked many questions about different foods such as cheese, milk, olive oil and coconut oil.  Engagement was high throughout the session, as everyone got involved in the activities and asked questions. The group were also very pleased to finally receive the pedometers (2 months late).  Engagement was high as all service users contributed to group discussions.  All service users participated in activities and discussions today.  Engagement was high amongst the group as they all participated in discussions and asked questions.  The group were engaged throughout the session, taking an interest and asking questions.  Engagement was high once again in today’s session, all service users participated in discussions and activities.  The group were all engaged and arrived early, as they were all eager/nervous about getting their blood results.  Good participation amongst the group today with many asking questions and engaging in discussions and group activities.  Service users were involved in the group activities and discussions, actively asking questions.  Engagement was very high again amongst the group, with each service user contributing to group discussions and activities.  Engagement was high amongst the group for the final session. |
|  |  | Site 2 = 6 | Engagement seemed higher this week compared to last week – the room felt a bit cooler which may have been a factor. A number of service users were taking notes and asking questions.  There seemed to be a high level of engagement with participants as they asked many questions about the fat content of different foods.  For the final part of the session the group did a quiz on what they have learned so far from the sessions. Engagement seemed quite high for this quiz.  Good group participation – all group members tried the exercises and took part in group discussions.  Engagement was high amongst the group as they all participated in group discussions.  Engagement was very high amongst the group, as each service user asked [Coach4] lots of questions and some stayed behind to ask questions too. |
|  |  | Site 3 = 3 | Engagement seemed high, many service users actively asked questions throughout the session.  Engagement seemed high, service users contributed to group discussions.  Lots of questions and engagement from the group today |
|  |  | Site 5 = 10 | All service users asked questions and got involved with the session.  Engagement seemed high as the group interacted throughout the session. They are getting to know each other more now and asking each other more questions.  Engagement seemed high – all service users asked questions and contributed to discussions.  Engagement was high, with the whole group contributing to the discussions.  [Coach1] even noted at the end that the ‘sharing stories’ activity at the beginning of the session was one of the best he’s had in terms of everyone contributing to the discussion.  Engagement was high amongst the whole group, as service users contributed to the discussions and clearly felt at ease to do so.  Engagement was high amongst the whole group, each service user contributed to the discussions.  Each service user participated and contributed to the discussions and were at ease.  Engagement was very high throughout the session, all service users contributed to discussions and worked in their groups. They were all at ease with each other  Engagement was very high again amongst the group, despite the groups being merged.  Service users were required to work in small groups for a number of activities during this session and they all contributed with the activities and discussions.  Engagement was high amongst the whole group as all service users contributed to discussions and asked questions.  Each service user appeared engaged in the group discussions and contributed in the activities. |
|  |  | Site 6 = 8 | Engagement seemed high throughout the session as members of the group were contributing to discussions.  Engagement was high as all service users contributed to discussions and asked questions.  Engagement was high amongst the group – everyone seemed to get involved with discussions and [Coach1] made sure to include everyone too.  Engagement was high throughout the whole session – all service users contributed and asked questions. Many service users also bought food packaging to look at as today’s session covered food labelling.  Engagement was high as all six service users took part in discussions and fed back their progress and goals.  Engagement was high as all group members participated in activities and group discussions.  All group members participated and actively asked questions.  [Coach2] just spoke for the first half hour of the session about the importance of making healthy lifestyle choices…However, service users engaged in conversation with her and asked her many questions throughout the session. |
|  |  | Site 7 = 1 | Group members were very willing to contribute questions and ideas; due to the smaller size, some may have found it easier to talk more/dominate the conversation a little |
| Mindfulness activities (n = 3) | Site 2 | Site 2 = 3 | Mindfulness was explained as a way to overcome internal barriers. The ‘Slowing down exercise’ was practiced amongst the group.  Note that this activity was very popular with the group and some service users asked to do this activity again at the end of the session so [Coach3] did the whole activity again.  Group do a 10-15 minute body scan exercise again. This was longer and a slightly different script compared to the last session.  The group reported to really enjoy this and felt relaxed.  Discussion about ‘What was important for me today?’  One service user stated to find the ‘raisin exercise’ to be really beneficial and another one stated that they enjoyed the body scan exercise. |
| Visual activities (n = 1) | Site 5 | Site 5 = 1 | Activity – [Coach1] described the pre-diabetes story using the magnetic man – he explained what happens when we consume glucose (healthy man) and what happens when we have too much glucose and pre-diabetes and Type 2 diabetes develops (unhealthy man).  The group reported that this activity helped them to understand the biology behind pre-diabetes a bit more. |
| Service user satisfaction (n = 12) | Sites 2, 3, 4, 5, 6, 8 | Site 2 = 2 | Note that at this point the service users reported that they had found these weekly sessions very useful as they learned a lot about foods and the right way to eat.  Note that service user reported that these group sessions exceeded her expectations and she has been “singing its praises.” |
|  |  | Site 3 = 1 | In the next group session taking place after this group, I asked the participants just coming in what they thought of the course and they were very positive – said the most useful thing had been what they had learned about what to do and what not to do |
|  |  | Site 4 = 2 | One woman had been coming for “near enough two years for one reason or another”  This woman was very, very positive about the programme and said she told lots of people about the course  New service user reported during consent taking that he’d found the core sessions of this programme really helpful, made a big difference to being “regular in the morning” etc. with all the information about fibre, wholemeal etc. Had already lost weight due to illness with COPD, but had had help with breathing exercises which had helped and now doing much better |
|  |  | Site 5 = 4 | At the end of the session (session 1) [Researcher] heard service users talking to each other on the way out saying how they enjoyed the session much more than they thought they would.  A service user noted at the end of the session that this session (maintenance 6) has been really useful for her and helped her to understand her eating behaviours.  One service user reported to [Researcher] that he thought [Coach1] was brilliant, and he thought that the diabetes prevention groups were also great and believed that this should be made available to everyone (if there were the funds!)  Discussion about having different levels of motivation…One service user stated that this session has really helped him to see what changes he can make. |
|  |  | Site 6 = 1 | One lady said she has found the sessions amazing |
|  |  | Site 8 = 2 | One man came over to re-introduce himself to [Researcher] and say thank you to [Researcher] and [Coach4], said it was “a great session” (referring to the whole programme I think)  The service user gave very good feedback on the programme, she said she hoped it would continue and that everyone would get as much out of it as she had; she said the main thing she had learned was knowledge about what to eat and what to avoid |
| **Category 2: Good group relationships (facilitator and peers) [n= 51 instances]** | | | |
| Good rapport (n = 27) | 1, 2, 4, 5, 6, 7 | Site 1 = 5 | Throughout the session [Coach] addressed service users by their names and it was clear she was making an effort to get to know them.  Clear that [Coach] makes the effort to get to know all the service users as she addresses them by their name.  [Coach] has developed a good rapport with service users and it is clear that she is a people person. She makes an effort to remember everyone’s names.  [Coach] welcomed each service user as they arrived and asked them how they were.  [Coach] welcomed each service user by name as they arrived and she chatted to them – clearly a good rapport has been built. |
|  |  | Site 2 = 5 | [Coach1] seems to have a good rapport with service users and they seem happy to approach her with questions, either before/after the session or during the session itself.  The group seem to have established a good rapport with [Coach1].  As service users arrived, [Coach4] greeted each of them individually, he remembered them all (e.g. he asked one lady how her operation on her foot went).  It was clear that the group really enjoyed the sessions with [Coach4] (and it was clear that he enjoyed delivering the sessions too).  Some of them were stating that they hoped it would be [Coach4] delivering their final one-to-one session in two months’ time.  At the end of the session [Coach4] told the group what a lovely group they had been and it was clear that they have enjoyed him delivering the final two sessions.  Note that this group also met [Coach4] during core session 2 when he delivered their physical activity session.  [Coach4] was very warm and friendly when the service user arrived for her consultation, and he remembered her from running their maintenance sessions.  Clear that he has built a good rapport with service users in the area. |
|  |  | Site 4 = 3 | Asking everyone’s name and writing it on the board was a good way of cementing the introduction and relationship with the group, made everyone’s name and presence important  Confident and knowledgeable, good rapport with participants  Positive comments from service users about how well they get on with the facilitator and how good she has been |
|  |  | Site 5 = 10 | [Coach1] was very engaging and welcomed each service user individually as they arrived. He also went down to reception to make sure everyone could find their way to the room.  [Coach1] was very engaging in the way he delivered the session. All the service users got involved with the discussion and asked questions. [Coach1] seemed to build a rapport with the group very quickly.  [Coach1] was very engaging and welcomed each service user individually as they arrived. He remembered each individual as he asked them relevant questions relating to the previous week.  [Coach1] was very engaging in the way he delivered the session – he asked many open questions and had already built a good rapport with each service user, even though its only session 2.  [Coach2] had already remembered some names of group members, despite this not being “her” session and despite only meeting them for the first time last week.  [Coach1] welcomed back each service user – the group was merged with different groups today and [Coach1] still remembered everyone’s name despite not seeing some of them for 7 weeks after being on holiday.  He asked each service user how their summer had been and took an interest in what they had been up to – “What does your son do? When does he go back to school?”  [Coach1] welcomed each service user as they arrived to the group.  He remembered each service user and addressed them by their name as they entered the room, despite the maintenance groups all merging together.  All service users seem to really like [Coach1] and they all give positive feedback about him to [Researcher] during the sessions.  [Coach1] has clearly built a good rapport with all service users.  [Coach1] remembered everyone as they arrived, including small details like “how did your operation go last month?”  It is clear that [Coach1] has developed a good rapport with the group as they were all at ease and made jokes with him.  One lady even bought him a homemade curry for him to have to dinner.  [The group] were all at ease with each other and made light-hearted jokes with [Coach1] throughout the session.  He remembered each service user by name, even though they are all from different groups.  One service user reported to [Researcher] that he thought [Coach1] was brilliant, and he thought that the diabetes prevention groups were also great.  [Coach1] welcomed each service user as they arrived to the group and congratulated them on getting to the end of the programme. |
|  |  | Site 6 = 3 | Already [Coach1] knew the name of service users and made an effort to address each of them by their name.  [Coach1] has got to know service users’ names now and addresses each with their names and involves everyone in the group discussions.  [Coach1] addressed each of the service users by their name. |
|  |  | Site 7 = 1 | [Service user feedback] They both agreed that the previous facilitator [Coach2] had been excellent, came in prepared, knew what she was going to do and did it |
| Tailoring the intervention to Asian ethnicities (helps to build good rapport?) (n = 1) | Site 4 | Site 4 = 1 | South Asian ethnicity (based on researcher judgement and [Coach1] said she speaks Gujarati), which was useful for recognising and spelling participants’ names on the flipchart sheet; [Coach1] told the group in response to one woman that she understands Gujurati if the woman needed to say anything in that language |
| Grateful to facilitator (n = 5) | Sites 1, 3, 5, 8 | Site 1 = 1 | All service users thanked [Coach] for her continued support over the 9 months. |
|  |  | Site 3 = 1 | At the end of the session several participants made a point of thanking the facilitator as they had found the course really useful, and [Coach7’s] support in particular; they said the most important thing for them had been increased awareness (about diet, exercise, diabetes risk) |
|  |  | Site 5 = 2 | The group were also sad to hear the news that [Coach1] would no longer be delivering the group sessions (they all let out a sigh), but also all congratulated him on his new role, with one service user saying “shall we all go to the pub and celebrate?”  At the end of the session they all gave him a round of applause and thanked him for the group sessions. It was clear that [Coach1] was well liked and respected by all service users.  At the end of the session, service users sang their praises for [Coach1], one lady gave him a thank you card and a bottle of wine (final session). |
|  |  | Site 8 = 1 | Group applauded [Coach4] at the end of the class, said thank you for the group; everyone chatted as they left, saying nice to meet you, good luck, etc. |
| Facilitator delivery style, knows session content (n = 4) | Sites 3, 6, 8 | Site 3 = 2 | [Coach 5] Shared a lot of personal anecdotes with the group which went down well, e.g. about own diet, history with food, cooking methods  One service user approached me at the end to say he really enjoyed this facilitator [Coach 4] and his style; when asked why he said he’s obviously knowledgeable, knows what he’s talking about, is lively and interesting, and the “first girl seemed to be just reading it out”; I briefly fed this back to the facilitator after everyone had left and he was pleased to hear this |
|  |  | Site 6 = 1 | One man stated that he liked [Coach2] because she could answer any questions that were put to her and he has been in previous groups where coaches have hesitated at questions. |
|  |  | Site 8 = 1 | Some attendees stated [Coach1] was “very good”, “informative”, “a good lecturer” – interesting they see her as a lecturer (there to provide education) |
| Group support (n = 14) | Sites 1, 3, 4, 5, 6, 7, 8 | Site 1 = 3 | It is clear that the group get on with each other and are now supporting each other. One lady talked about not losing any weight this week and the rest of the group gave much positive reinforcement and discussed solutions to triggers.  [Coach] asks: “How is everyone feeling now that we’re near the end?”  Discussion that service users in the previous group have agreed to meet for coffee once a month to continue supporting each other.  At the end, some service users write down their contact numbers and make arrangements to meet up in one month’s time on Tuesdays to continue supporting each other. |
|  |  | Site 3 = 3 | The group were good at giving each other suggestions today, e.g. if one had a question the others gave answers, such as “Try All Bran, it has lowest sugar for a high fibre cereal”  The service users are now very chatty with one-another, they’re very open about giving advice and asking questions of each other – good group relationship for peer support  One participant is usually brought by her daughter who acts as her carer, but the daughter will be unable to bring her for the next few groups so the participant would have to miss them; another group member offered to collect her and drop her off so she can still attend, and they swapped phone numbers; suggests camaraderie between the group |
|  |  | Site 4 = 4 | Group were chatting to one-another before the session started, appeared friendly with one-another  One lady helped others complete their forms and comforted bereaved woman next to her when she became upset  Lots of group support/discussion; understanding, empathy, suggestions, honest accounts and experiences; e.g. “that’s good!”; giving suggestions about what types of food to eat to each other  She also mentioned the group could swap contact details and support each other if they wished  Facilitator mentioned previous groups a few times to give them positive examples to follow (e.g. supporting each other after the end of the course) |
|  |  | Site 5 = 1 | [The group] Seemed quite relaxed and friendly with one-another, quite a few jokes made and chatting to one-another |
|  |  | Site 6 = 1 | It was clear that service users (especially those from the original cohort who had been in the same group since the beginning) have developed a good rapport with each other and support each other. |
|  |  | Site 7 = 1 | While one-to-ones happening the group were discussing both on-topic and off-topic things, e.g. foods they had been eating, where they were eating, decisions they had made, their progress on the course; but also holidays, where they lived, the local social club, local people  When people came back to the group from their one-to-one the group members would ask how they had gotten on, and the person usually reported the change in their blood result, their reaction to this, and a qualifier, e.g. “but I still need to work on my waist circumference”, or “but I don’t know how I managed that” |
|  |  | Site 8 = 1 | The group works well together, good relationships between service users, good peer support (e.g. congratulating each other if lost weight at start of the session) |
| **Category 3: Service user behaviour change [n = 17 instances]** | | | |
|  | Sites 2, 3, 4, 5, 6, 7, 8 | Site 2 = 1 | One service user also reported to [Coach1] during the 3-month review that [Researcher] observed that he had started exercising more after attending last week’s session and he had started going for longer walks which resulted in him feeling more energised. |
|  |  | Site 3 = 2 | One service user said he was tracking his weight every day and had brought in a graph of his progress over the course; graph showed daily fluctuations, but an overall downward trend; facilitator used the graph to emphasise to the group that weight fluctuates so often better to measure weekly or take an average, but if it works for some like the person with the graph that’s good/fine  One man said he had lost 9kg and his family commented on how much weight he had lost, but he felt very healthy and strong; one man said he would carry on with what he had learned, as he had been encouraged to do more exercise; he had made most changes in the first period of the course, but had managed to maintain it |
|  |  | Site 4 = 4 | While waiting, [Researcher] asked the service user how he was finding the class – he said very good as his natural motivation to change was very low without outside influence, but just in the space of a week he had lost a couple of KGs and was very impressed and please with this; now that he was on the course, felt he had to take it seriously; had been more conscious of what he was eating; choosing smaller/fewer portions; discussed making small changes over long term making a big difference, not cutting things out entirely, can still have the occasional treat, etc.  The man attending with his wife said they were making changes together so it makes sense for him to come along and learn too  Started off by saying how very pleased she was as everyone or 99% of them had lost weight, which she wasn’t expecting over Christmas, but they had done really well – followed by a group round of applause  Chatted to a participant beforehand who said he had learned “lots of small things”, was very positive about the programme, had stopped having sugar in his tea and coffee, followed the suggestions except on occasions such as visiting his grandchildren, and thought everyone should be taught these things, advocated teaching about diet in schools |
|  |  | Site 5 = 1 | At the start of the session, [Coach1] gave an introduction to the final session and the group discussed their biggest achievements and changes they have made since being on the programme.  Changes included improved diet, more exercise, increased awareness and a better quality of life.  [Coach1] reported that the whole group had reduce their blood sugar levels which is “fantastic.” |
|  |  | Site 6 = 1 | One lady said she has found the sessions amazing, and since starting on the programme last year she has lost 2 and a half stone. She reported to have learned so much from the course. |
|  |  | Site 7 = 7 | One woman had managed to do 8,000 steps every day this week, had even done 13,000 one day, and had walked 45 minutes home from the shop one day – sees the group as worthwhile; only 66, doesn’t want to be resting and elderly yet!  Another woman was discussing with [Researcher] how she had gone from a size 20 to a size 14, and was in very bad health but now mobile, although she still suffers with very high blood pressure and other health problems  While person having their one-to-one the other three were discussing their surprise at having lost weight according to differences in their clothing, e.g. one man had to have his watch resized as it was now too big for him; one woman had come to see [Coach2] separately to catch up as she had missed a few sessions and was surprised she had lost 7cm around the waist; the third person had also lost circumference around the middle and “felt slimmer”, so they concluded “everybody’s doing well”  One woman chatting to [Researcher] said she doesn’t enjoy preparing meals, but has learned lots of new recipes after coming on this programme (e.g. had never thought to use a tin of chopped tomatoes until now); now thinks about meals over the week as a whole and not day by day; has more vegetables despite her husband really complaining and disliking them; feels she is taking this “seriously” and is doing well; thinks the booklets on positive mental wellbeing are very relevant and discussed that she can sometimes have very poor sleep, but it’s unpredictable  One woman was particularly surprised at her blood glucose results and had steeled herself for “bad news” – was “shocked” it had come down to the normal range; [Coach2] suggested maybe she had been making more changes than she realised and to try and keep this up, not to become complacent now she had had a good result  One person said she really didn’t want to get diabetes as her friend had type one and she saw the effect it had on her life, so she had made a lot of changes, taken on the course wholeheartedly (although it was a “bit of a drag”), was cooking different things which had been quite fun, and while her partner was often sceptical, he quite enjoyed the meals; she had managed to lose weight and waist circumference, and brought her blood glucose down into the healthy range; she did not point out that losing weight around the middle was expensive in terms of new clothes, and that you lose weight around your face which people notice and ask if you’re okay (looking gaunt)  Asked both of these women about what they thought of the length of the programme and frequency of sessions; one said it had to be a year in order to see changes in the HbA1c results |
|  |  | Site 8 = 1 | The service user had lost a lot of weight before the programme had even started, after she had been told she was “pre-diabetic”; her partner had researched diets and they had been following a high fat, low carb approach with the occasional treat; this was working for them and her partner had lost three stone while she had lost one stone; she was concerned in case her weight had crept up a few pounds, but on hearing today’s weight she was happy it was exactly 9 stone, so she is maintaining the weight she’d like; she also didn’t want to lose much more weight as she would start to look poorly |
