# Supplementary material for: The NHS Diabetes Prevention Programme: an observational study of service delivery and patient experience
Source: BMC Health Serv Res. 2020 Nov 27;20:1098. doi: 10.1186/s12913-020-05951-7 (PMC7694420; doi:10.1186/s12913-020-05951-7)
Supplement: Supplementary file 3 — Additional file 3:. Negative patient experiences, extracted from observational notes. [file 12913_2020_5951_MOESM3_ESM.docx]

**Additional File 3. Negative patient experiences extracted from observational notes, corresponding to each category**

| **Sub-category** | **Sites** | **No. of instances observed** | **Extracted from observational notes** |
| --- | --- | --- | --- |
| **Category 1: Scheduling and size of group sessions [n = 41 instances]** | | | |
| Incorrect or changed session date / time (n = 6) | Sites 1, 3, 4, 7 | Site 1 = 2 | At the beginning of the session service users were asking about the dates of future sessions, as one service user had her list of dates that was sent in the post which stated that there was a session on 1st January, but everyone else had the 8th January. [Coach] confirmed there is no session on 1st January but will go over future dates of sessions next week.  Note that other service users could be sent the wrong dates through the post in error.  Before session started, one lady told [Coach] that she came to a session on the 7th May (4 weeks after previous session) but the session was actually on 30th April (3 weeks after previous session).  [Coach] apologised that this lady’s letter had the incorrect dates on it.  There was apparently 4 people who had turned up on the wrong date. |
|  |  | Site 3 = 2 | Mix up with appointment times; one woman had arrived for an appointment at 9.45 but [Coach1] wasn’t scheduled to start until 10am; woman was very annoyed and had to leave (left her name and number with reception); said she wouldn’t bother trying again with DPP  Fewer people than usual this week (6 at the start of the session, plus family member, plus one service user arriving late – usually around 11 people)  Before recording and start of session facilitator went to check at front desk if anyone else arrived; found out from receptionist that a lot of people turned up last week and were upset they’d been given the wrong date  Facilitator thought they’d been sent a letter with the wrong date, whereas the people in the room said they’d been sent a text message the day before as usual |
|  |  | Site 4 = 1 | One man had been told the group was at 9.30am so turned up two hours early, when the group actually started at 11.30am (poor communication) |
|  |  | Site 7 = 1 | Time had been changed the day before the session from 2.30pm to 2pm and the facilitator [Coach2] had been unable to get hold of a few people, so there were only 4 people attending  Because of this, [Coach2] decided that the content she would have covered (the module) would instead be delivered at the January session when more people were present. |
| No text reminder for session (n = 5) | Sites 1, 2, 3, 7 | Site 1 = 2 | Note that service users stated that they had not received any text reminders for this session.  [Coach] stated that she thinks there are people off in the office this week which may explain why they have not received a reminder text.  Service users were no longer receiving text reminders. [Coach] was also going to inform the office that there are no longer text message reminders being sent to service users which may also help in future. |
|  |  | Site 2 = 1 | Before the session began [Researcher] overheard some informal conversations between service users – one was saying that he was grateful for having a text reminder for the sessions, but another lady stated that she had never had any text reminders.  Interesting to note that this may be a possible reason why some may not attend the maintenance sessions if they have forgotten. |
|  |  | Site 3 = 1 | Problem with text messaging system means people not receiving reminders; the last texts sent were sent directly by [Coach6], not by the automated system; [Coach6] had been in touch with everyone who had missed the last couple of sessions |
|  |  | Site 7 = 1 | Some people mentioned the text messaging system was not very good; the new facilitator [Coach2] said she doesn’t use this at all, so won’t be a problem from now on. |
| Unable to confirm session dates (n = 4) | Sites 4, 6 | Site 4 = 1 | Another participant requested a schedule of all the dates in advance; again [Coach2] said she would raise this with the company as they weren’t the first to request this |
|  |  | Site 6 = 3 | Unsure when the next session is, however one service user stated that she called up to complain that they were not given dates in advance (could not work around work commitments etc) and she said they told her about sessions scheduled for August and November.  One man said that he has also enjoyed the course, but the scheduling of sessions has been very unorganised (not knowing session dates).  Lady said that she has enjoyed the course, but there has been some real issues with the scheduling of sessions and being given short notice for review sessions. She was unable to attend her session 11 which was 3 weeks ago, and [Provider] told her not to reschedule the session and just come to session 13 instead. |
| Merging of group cohorts in maintenance sessions (n = 3) | Sites 2, 4 | Site 2 = 1 | Service user suggested that the core and maintenance groups should remain the same rather than merging different groups together for the maintenance sessions. [Coach4] explained that this was due to drop-out rates. |
|  |  | Site 4 = 2 | While waiting on a taxi chatted to one woman about how most people from her original group had dropped off and there were people there tonight she hadn’t seen before, but she and one other man stick together and come to same groups so they can do it together; [Researcher] mentioned 6pm group moving around as follow up session not booked.  One woman said she really enjoyed the programme and coming for the weekly core sessions, but then there was a huge drop-off in support when it moves to monthly, especially when they can’t attend some sessions due to the schedule changing the time from 6pm to 4pm when working people can’t attend; this was the first session in the new year she was able to come to for that reason (started attending core sessions in July); she wants to come, finds it important as her parents had been diagnosed with diabetes and the health improvements he was making were spreading to her family; however she’s been unable to come due to the session timing; she takes notes and can refresh her memory, but it still means lost momentum; the call centre staff are very good and understanding, but can’t help; she liked coming along with the same people for weekly sessions as they got to know each other and spoke to each other, now it’s always different people; says this is her “only gripe” (would prefer weekly support and more evening sessions); telling [Researcher] this as [Researcher] had said we’re evaluating the programme and she wanted to feed this back |
| Double booking of IA’s (n = 2) | Sites 3, 7 | Site 3 = 1 | Other attendees had come for appointments before 10am, some were double booked. |
|  |  | Site 7 = 1 | Participant had previously been given the wrong date to attend for his IA, so had arrived when a group was running last week; coach apologised for this; participant didn’t seem perturbed |
| Cancelled sessions not communicated (n = 4) | Sites 3, 4 | Site 3 = 1 | Two people (one woman alone, and one man bringing his elderly mother from her care home in a wheelchair) turned up for the 1pm group which had been cancelled due to low numbers – call centre had contacted them to reschedule them; facilitator not good at communicating with these unexpected arrivals, researcher had to say “think it’s been cancelled” before [Coach1] checked their name on attendee list and advised them to contact the call centre with the number on their letter/email; when the second woman arrived she called her co-ordinator and advised the woman to come next week to either the 10am or 11.30am group |
|  |  | Site 4 = 3 | [Researcher] arrived early for 6pm class around 5.40-5.45, but [Coach1] not present  Two service users arrived, waited until 6pm then [Researcher] text [Coach1]  [Coach1] replied to apologise, she had cancelled the session due to personal circumstances by informing the coordinator but had not remembered to contact [Researcher] due to stress of the situation  One especially was very annoyed as she had missed an important event in London to be there  [Researcher] advised they call the call centre to inform them that they had not been told of the cancellation, unlike the rest of the group who had known not to turn up  The more annoyed service user copied down the phone number from the other service user as she had just thrown away the original letter  One participant complained to coach 1 that she hadn’t been contacted last week when the class had been cancelled – she had missed an important trip to London and an audition for this, then the class wasn’t on; the coach explained she had told her coordinator and the woman should have been told, but was very sorry she had not been informed  Note: [Researcher] attended [Location] on Monday 1st April after checking on Friday 29th March by text that the session as running; on arrival discovered the maintenance session had been cancelled in favour of a core session (two core sessions combined, to be delivered by another experienced coach with [Coach2] present as she had just gotten her voice back after an illness); several participants had also arrived for the maintenance session and had to be turned away |
| Unable to book onto sessions (n = 1) | Site 4 | Site 4 = 1 | While waiting outside the room for an earlier group to leave, there was chat among service users about poor organisation and management of the programme  One woman had called up to book next classes, one was oversubscribed so she couldn’t go; one was in [Location] so that was no use for her |
| Waiting list to get onto course (n = 3) | Sites 1, 4, 7 | Site 1 = 1 | Said he had been “trying to come on the course for ages” ever since receiving the high blood glucose reading; finger-prick HbA1c test revealed his blood glucose was now in the normal range;…course was not offered. |
|  |  | Site 4 = 1 | While waiting outside the room for an earlier group to leave, there was chat among service users about poor organisation and management of the programme…Other woman waited 9 months to get on the programme, and frustrated that now she can’t keep going as there’s no equivalent follow up date (i.e. at 6pm, this venue); she had called for dates and had wanted a class at the hospital, but no one called her back, twice! |
|  |  | Site 7 = 1 | [Researcher] asked them how long they waited between IAs and this group; one woman said not long (letter received while on holiday and missed the start of another group), but the others (3 or 4 women) said it had been a long wait, from one month to several months, and some said equally long wait between receiving a letter about high blood glucose and the IA  Seemed as though most if not all had been invited via letter |
| Large gap between maintenance sessions (n = 2) | Site 4 | Site 4 = 2 | While waiting outside the room for an earlier group to leave, there was chat among service users about poor organisation and management of the programme…One man had attended four core sessions in June, has had a long gap, now back for maintenance 4-5 months later, said he “knows you can do them any time in any order”  Other man had been to three or four maintenance sessions already, so was doing them out of order  This service user and others’ core sessions were six months ago!  Another service user had switched venues so that he could carry on attending without a large gap, as the next ones available in [Location] were not until March (?); he finds attending the sessions and knowing he will be weighed very motivating for lifestyle change, otherwise he might just let himself away with it!  The session involved asking service users to remember content from core sessions, however for many of them this was so long ago it was difficult to remember and [Coach2] recapped the content for them |
| Lack of notice for sessions (n = 1) | Site 6 | Site 6 = 1 | Two service users complained about the lack of notice for this review session – one lady was only given notice at 5:30pm yesterday afternoon and another man was given notice at 9pm yesterday evening and he had to cancel some plans in order to attend the session today. |
| Issues getting through to call centre (n = 2) | Sites 4, 5 | Site 4 = 1 | Also reported lots of problems with the call centre as many people are not able to get through to speak to someone |
|  |  | Site 5 = 1 | Note also that when discussing the booking of maintenance sessions, one man raised the issue that when he has tried to call the call-centre he hasn’t been able to get through for hours and that the man he spoke to on the phone he said that he was the only one in the call centre.  [Coach2] stated that they have since expanded their call centre but it is also best to call early morning to get through quickly.  This could put people off attending maintenance sessions if they have to call up to reschedule a session they cannot make? |
| Group size: Many people in attendance (n = 6) | Sites 1, 3, 4 | Site 1 = 4 | Service users were very unhappy at the beginning of the session and were quick to comment that too many people had been booked onto the group. Some said would not be returning because it is too unorganised. However, by the end of the session they seemed to have enjoyed it and were looking forward to the next session.  There were 17 service users in attendance – as there were a lot of group members today, they couldn’t all fit around the table so three service users sat on seats away from the table.  There was a high turn out today (n=17) so not everyone could fit around the table in the centre of the room. 4 people sat on chairs around the edge of the room.  The room was full today so about 5 people were sitting around the edge of the room and the rest of the group were sitting around the table. |
|  |  | Site 3 = 1 | Not enough chairs laid out at the start of the session (a large group), one woman had to get her own unprompted from a tall stack of chairs. |
|  |  | Site 4 = 1 | Difficult to manage the group with so many people attending; had to split the group into two for two activities, however even half the group couldn’t all fit around the activity table where [Coach2] had laid out meal plate photos and calorie cards to match how many calories per meal; lots of talking so difficult to hear all of the conversation and not everyone gets a chance to join in; the other activity where discussing ways of increasing wholegrain/fruit and veg/reducing salt was slightly better as didn’t all need to see a visual activity |
| Group size: Less people than usual in attendance (n = 2) | Sites 3, 8 | Site 3 = 1 | Only 4 patients and one care attending today; one participant quite concerned about this and asked the coach twice to make sure the others were contacted to attend the next session [note that previous session was cancelled] |
|  |  | Site 8 = 1 | Group commenting prior to start of class about how numbers have dropped from the beginning of the course, now only 8 or 9 people after starting with over 20 |
| **Category 2: Factors influencing disengagement / dissatisfaction within the session [n = 27 instances]** | | | |
| Lack of engagement / sleeping (n = 2) | Site 2 | Site 2 = 2 | Session was based on [Caoch1] talking. Looking around the room, especially during the second hour, there were at least 3 participants who were sat with their eyes closed, so engagement was low for some (although the room was very hot). On the other hand there were a select few who were taking notes and answering questions.  There was one lady who kept falling asleep and then also answered her phone in the session during the final activity. Though important to also note that the room was very hot. |
| Too much technical information / complex health messages (n = 3) | Site 8 | Site 8 = 3 | In this session there was a lot of information, some quite technical, given in very quick succession; keeping up with the content was aided by following the content in the handbooks; one service user at the end said it was “too much”  Some confusion over aims of activity, e.g. low GI is healthy  Not much clarification or explanation of relationship between messages about low GI and how this fits with other health messages, despite questions from participants  Gave some quite complex information, e.g. “1,7 mmol of triglycerides”  This session was very heavy going – for over an hour there was information about very serious health consequences and risks of type 2 diabetes, with no activities to break it up; by the time they had a break people were commenting on “brains bursting” |
| Difficult activities (n = 1) | Site 2 | Site 2 = 1 | To begin session 3, [Coach1] then asked the group to work out the number of portions of carbohydrates they had eaten based on their self-reported food diary that they had just filled out for the previous day.  There were instructions for how to work this out in the participant handbooks – there was a table detailing some foods and their number of carbohydrates.  The group seemed to find this activity hard, as the participant handbooks did not list all of the foods they consumed so they had to look up a lot of the carbohydrate content of their foods on the internet (or [Coach1] was looking it up on her phone for them). This meant that the activity took a long time to complete (until 2:50pm, session started at 2pm).  A lot of individuals were asking [Coach1] for help – e.g. “How much carbohydrates would be in a handful of nuts and berries?”  A few participants were waiting around for the next part of the session to start, some seemed to be sitting around for about 20 minutes. |
| Poor group management / poor engagement (n = 1) | Site 7 | Site 7 = 1 | [Coach1] had some trouble controlling chat in the group; some individuals were talking loudly to each other while trying to go round the group introducing each other in an ice-breaker activity; one of the other participants intervened and asked them to be quiet |
| Layout of room (struggling to see flipchart) (n = 4) | Sites 3, 8 | Site 3 = 3 | There was flip chart at the front of the room – some service users struggled to see the flipchart due to the arrangement of the tables.  Room set up with four long tables in a row and chairs behind; room very big so travel did not carry well, flipchart at the front hard to see as far from participants at the ends of the row  Flipchart placed far away from participants so difficult to read what was being written |
|  |  | Site 8 = 1 | One older lady in particular found it very difficult to see the flipchart; [Coach1] moved the chart as close as possible and indicated items in the book instead |
| Materials: Unable to provide resources (n = 6) | Sites 1, 2, 3 | Site 1 = 2 | [Coach] is still waiting for an order of the second batch of workbooks (9-12) so some service users had to share today but [Coach] said she will have them by the New Year.  There were 3 service users who have not had all of their workbooks yet and they asked [Coach] if she had bought them along to the session today, but [Coach] forgot that there were still some service users who didn’t have the workbooks so she promised to bring them for the next session in one month’s time. |
|  |  | Site 2 = 3 | [Coach1] only bought a few participant handbooks with her today as she said they were “too heavy to carry” so she will bring some more next week. This meant that only about a third of participants had received their handbooks in session 1. These handbooks would have clarified more about the diabetes story and the goal setting activity at the end.  [Coach1] bought a few more participant handbooks to the session today, however, there were still a handful of people without them. She is bringing the last few handbooks with her next week.  [Coach1] was due to bring the final batch of participant handbooks today (she stated that she couldn’t bring them all at once as they are heavy). [Coach] was reminded by a number of participants that they didn’t yet have their handbooks. However, she admitted that she forgot to bring the handbooks today so those participants now have to wait for 2 weeks to get these books as there is no session running next week due to a venue booking issue. This means that some participants would have waited 4 weeks for their handbooks. |
|  |  | Site 3 = 1 | Not many service users had a pen to complete this activity (or subsequent activities) and [Coach1] only had one pen to lend out. [Researcher] lent some pens to service users to complete the activities. |
| Materials: Issues with pedometers (n = 3) | Site 1 | Site 1 = 3 | It took a few minutes to explain to the group how to use them [pedometers].  Some had difficulty opening up the pedometers to read the screen.  Some pedometers seemed to be faulty as they would not re-set so [Coach] took those ones back in.  Some service users had not been recording their step counts in their lifestyle logbooks, and one stated that his pedometer was not working.  Note that some service users had been struggling with the pedometers as they were not accurately recording their steps. [Coach] helped these people to estimate their weekly steps.  [Coach] apologised for the “rubbish pedometers” but made the point that if the pedometers were encouraging people to do more steps/activity, then it may be worth investing in a better pedometer. |
| Materials: Not enough resources (n = 1) | Site 3 | Site 3 = 1 | Could do with more resources – facilitator having to borrow cards back from the group to do activity feedback; only enough resources for one between three service users |
| Service user feedback / dissatisfaction (n = 6) | Sites 3, 4, 6, 7 | Site 3 = 1 | During a task, one man said to [Coach4] he didn’t know why he was here, felt it was a waste of everyone’s time, so [Coach4] said it was up to him and let him go – think he was possibly a bit upset (watery eyes and other service user said “no point being here and getting upset” as he left), but [Coach4] may not have known or realised, and was quite short about him not needing to stay (no exploration of reasons why) – highlighted to me the importance of facilitators’ relationships with their groups and the need for continuity where possible |
|  |  | Site 4 = 2 | One woman said she would have like more demonstrations and practical sessions on e.g. cooking healthy meals  One man said he had switched venues as they had a new educator for the last couple of core sessions and he said she was “picked from somewhere” and “didn’t know what she was talking about”, “we probably knew more than her” (in front of [Coach2], who may have felt pressure from this!) |
|  |  | Site 6 = 1 | Note that the small room meant that service users could all hear (and watch) each others’ reviews. One lady made a comment to [Researcher] that it wasn’t very private and some people may feel sensitive about this. |
|  |  | Site 7 = 2 | Participant mentioned that more regular contact is needed, like earlier in the programme; at this point it’s very hard to keep self-motivated between visits when there is 3 months until the next contact  Both felt the programme had been somewhat disrupted due to changes in rooms, changes in facilitators, poor communication (e.g. it said 8 weeks, but was really 8 weeks plus the rest of the year) etc; also felt the later review sessions could be better as they were just sitting about while one person was seen; could have had individual appointments instead  They felt it wouldn’t have really mattered if they’d missed sessions as much of the themes were the same and they always had their books to look at  However they both agreed that the previous facilitator [Coach2] had been excellent, came in prepared, knew what she was going to do and did it |
| **Category 3: Venue [n = 15 instances]** | | | |
| Location: Access issues (n = 3) | Sites 1, 7 | Site 1 = 2 | …service users started to arrive and were asked to sit in the waiting room which only had 5 seats (all service users were elderly). They were directed to another small waiting room with some chairs, but this required going up a couple of steps which some service users may have found difficult.  One service user who uses a walker has to go outside and come back in through a side entrance to access the room as she cannot use the stairs…she has to ring the bell to be let back in again and then a member of staff has to unlock the door to let her out at the end of the session. |
|  |  | Site 7 = 1 | The room is on the lower ground floor, through the cafeteria, down some steps, near the gym, through a passageway making it somewhat difficult to find – one person in the group has some mobility issues and walks with a walking stick, not sure how easy this was for her to manage |
| Location: Venue far away from home (n = 2) | Sites 4, 7 | Site 4 = 1 | One woman asked if there were any classes in her local community centre as [Area] is hard to get to; [Coach1] explained the others available and that she could move her onto one of these for the maintenance sessions if she wanted (possibly not space to move her now), but none of the available groups were any easier for her to get to; she struggles with getting buses because she had had a stroke and didn’t have good balance; depends on her daughter collecting her |
|  |  | Site 7 = 1 | For one woman, attending the class is a “five hour round trip” as it takes two buses/ one hour to get there and get home |
| Temperature of rooms (n = 4) | Sites 1, 2 | Site 1 = 3 | [Researcher] had some informal chats with service users whilst waiting to enter the room – they were stating that the room is quite small for the size of the group and they found the room too warm without windows.  The room was very hot today but one service user managed to open the fire exit doors to get some air into the room.  The room was quite hot today so they opened the fire doors at the back of the room. |
|  |  | Site 2 = 1 | The room was very hot again, the air conditioning was adjusted throughout the session.  Note that the room was very hot again, some service users commented on this and [Helper] went to the reception desk to ask someone to turn on the air conditioning. |
| Noise issues (n = 2) | Sites 2, 3 | Site 2 = 1 | Service user suggested that she was not keen on the [GP surgery] venue (for her maintenance sessions) as the groups are held in the waiting room where some people can walk in and out and there are also loud beeps that occur when patients are called. But she did report liking the first venue (called the [Name] venue?) that she attended for her core sessions. |
|  |  | Site 3 = 1 | Room is very large and sound doesn’t travel well either for the recorder or for attendees to hear each other; |
| Venue / room hard to find (n = 4) | Sites 3, 5, 7 | Site 3 = 2 | Attendees said [venue] was hard to find (not well-known or well sign posted).  Some group members were a bit late as they had trouble finding the venue. |
|  |  | Site 5 = 1 | The group was delivered in a room in the Football Stadium. It was a different room again this week, instead of the usual room upstairs.  [Researcher] arrived at 17:20 and went up to the usual room. Some service users started to arrive from around 17:45. When [Coach2] had not arrived by 17:45 [Researcher] went downstairs to ask reception if there had been a room change. [Researcher] met [Coach2] downstairs who was informed about the room change – it was now in a room next door rather than a meeting room in the [Location].  [Coach2] was told that a man would escort the group to the room once everyone had arrived.  [Researcher] went upstairs to the original room to collect the service users already waiting there.  7 service users plus [Coach2] and [Researcher] were escorted to the room at 18:00 (4 arrived late due to not being informed of the room change and having to be escorted). |
|  |  | Site 7 = 1 | In a different room this week – right along the end of a very long corridor, through many double doors. A few people found the room hard to find. Said the notice board at reception wasn’t very clear. |
